# Seasonal variations in body melanism and size of the wolf spider *Pardosa astrigera* (Araneae: Lycosidae)

**DOI:** 10.1002/ece3.3988

**Published:** 2018-04-02

**Authors:** Jinjian Yang, Qijia Wu, Rong Xiao, Jupeng Zhao, Jian Chen, Xiaoguo Jiao

**Affiliations:** ^1^ Center for Behavioral Ecology & Evolution Hubei Collaborative Innovation Center for Green Transformation of Bio‐Resources College of Life Sciences Hubei University Wuhan China; ^2^ Guangdong Entry‐Exit Inspection and Quarantine Technology Center Guangzhou China

**Keywords:** Bergmann's rule, body melanism, fecundity, *Pardosa astrigera*, phenotype plasticity, seasonal variations

## Abstract

Variations in species morphology and life‐history traits strongly correlate with geographic and climatic characteristics. Most studies on morphological variations in animals focus on ectotherms distributed on a large geographic scale across latitudinal and/or altitudinal gradient. However, the morphological variations of spiders living in the same habitats across different seasons have not been reported. In this study, we used the wolf spider, *Pardosa astrigera*, as a model to determine seasonal differences in adult body size, melanism, fecundity, and egg diameter both in the overwintering and the first generation for 2010 and 2016. The results showed that in 2010, both females and males of the overwintering generation were significantly darker than the first generation. Moreover, the overwintering females were markedly larger and produced more and bigger eggs than the first generation in both 2010 and 2016. Considering the overwintering *P. astrigera* experiencing low temperature and/or desiccation stress, these results suggest that substantially darker and larger body of the overwintering generation is adaptive to adverse conditions.

## 1 INTRODUCTION

Variations in species morphology and life‐history traits strongly correlate with geographic and climatic characteristics (Ashton, Burke, & Layne, 2007). Body melanization is a common form of phenotypic variations in ectotherms, especially in insects (Majerus, 1998; Stoehr & Wojan, 2016; Umbers, Herberstein, & Madin, 2013). In addition to the contribution of melanism to cryptic coloration, ultraviolet protection and disease resistance (Majerus, 1998; Reguera, Zamora‐Camacho, & Moreno‐Rueda, 2014), other two hypotheses have been proposed for melanism: thermal melanism and melanism desiccation (Clusella‐Trullas, van Wyk, & Spotila, 2007; Davis, Farrey, & Altizer, 2005). According to thermal melanism, darker forms are more efficient in heating their bodies from the solar radiation than their lighter counterparts (Clusella‐Trullas et al., 2007; Davis et al., 2005; Kuyucu, Sahin, & Caglar, 2018). Therefore, darker individuals have an advantage in cold environments whereas the lighter individuals may have an advantage in warmer conditions. Variations in melanization with altitude and latitude are in agreement with the thermal melanism hypothesis, that is, darker individuals are frequently found at higher altitudes and latitudes, and lighter individuals are common at lower levels (Clusella‐Trullas et al., 2007; Kuyucu et al., 2018). Conversely, the melanism desiccation hypothesis states that melanism contributes to desiccation resistance, that is, melanic individuals have a high desiccation resistance, while non‐melanic individuals have a low desiccation resistance (Daniels, Mooneyk, & Reed, 2012; De Souza, Turillazzi, Lino‐Neto, & Santini, 2017; Gibbs, Fukuzato, & Matzkin, 2003; Parkash, Rajpurohit, & Ramniwas, 2008; Parkash, Ramniwas, Rajpurohit, & Sharma, 2008; Parkash, Sharma, & Kalra, 2010; Rajpurohit, Parkash, & Ramniwas, 2008; Ramniwas, Kajla, Dev, & Parkash, 2013).

Body size is another important life‐history trait, because it correlates with numerous physiological and fitness traits including fecundity and survivorship (Honěk, 1993). In insects, body size is a common form of phenotypic variation in response to the external environment, particularly the temperature, which has a direct effect on body size; body size increases as temperature decreases and vice versa (Atkinson, 1994; Chown & Gaston, 1999; Partridge & French, 1996). In addition to temperature, photoperiod could also affect the body size of some insects. In general, adults are larger when their offspring are reared under a long photophase than a short photophase (Nakamura, 2002; Niva & Takeda, 2003; Zerbino, Altier, & Panizzi, 2014, 2015).

Similarly, egg size in ectotherms commonly increases in colder regions and colder seasons (Azevedo, Partridge, & French, 1996; Blanckenhorn, 2000; Fischer, Bauerfeind, & Fiedler, 2006; Fischer, Bot, Brakefield, & Zwaan, 2003; Fischer, Bot, Zwaan, & Brakefield, 2004; Fischer, Brakefield, & Zwaan, 2003; Yampolski & Scheiner, 1996), and in laboratory conditions insects lay larger eggs at lower temperatures (e.g., Avelar, 1993; Blanckenhorn, 2000; Crill, Hucy, & Gilchrist, 1996; Ernsting & Isaaks, 1997; Fischer, Bot, et al., 2003; Fischer, Brakefield, et al., 2003; Fischer et al., 2004, 2006; Seko & Nakasuji, 2006). However, the mechanisms underlying the temperature‐size rule as well as its adaptive significance are largely unexplored (Azevedo et al., 1996; Blanckenhorn, 2000; Crill et al., 1996; Fox & Czesak, 2000).

In insects, fecundity often positively correlates with female body size (Honěk, 1993). It is predicted that the factors substantially affecting female body size may induce variations in fecundity. However, studies that focus on the fecundity variations due to female body size influenced by the environment (temperature) are limited.

Most studies that focus on the variations of morphology and life‐history traits are limited to ectotherms distributed on a large geographic scale, such as a latitudinal and/or altitudinal gradients (Azócar et al., 2015; Lack et al., 2016; Moriti, Nakas, Köppler, & Papadopoulos, 2012; Reguera et al., 2014; Tu et al., 2011; Tuomaala, Kaitala, & Rutowski, 2012; Valenzuela‐Sánchez, Cunningham, & Soto‐Azat, 2015). However, the variations of morphology and life‐history traits of the spider species living in the same habitats across different seasons are poorly explored (but see Edgar, 1971; Iida & Fujisaki, 2007; Iida, Kohno, & Takeda, 2016; Miyashita, 1969; Schmidt, Harwood, & Rypstra, 2013).

The wolf spider, *Pardosa astrigera* Koch, is a wandering spider widely distributed in East Asia (World Spider Catalog 2017). In most central provinces of China, two generations occur per year; the overwintering individuals emerge in August and mature in the following March and the first generation emerges in late March and matures in early July. The reproductive peaks generally occur in early March for the overwintering generation and in early July for the first generation. The overwintering sub‐adults are inactive from November to late February. In this study, we used the wolf spider, *P. astrigera*, as a model to determine the differences in body melanism, female and male body size, fecundity, and egg diameter between the overwintering and the first generations in two different time periods. The results reported here provide insights on the adaptive consequences of body melanism and body size to seasonal variations for the wolf spider, *P. astrigera*.

## 2 MATERIALS AND METHODS

### 2.1 Spider collection

In 2010, adult female and male *P. astrigera* of the overwintering generation were collected in late March and the first generation was collected in late July from Ma'anshan Forest Park, Wuhan, Hubei Province, China. In 2016, adult females and males of the overwintering generation were collected from the same site in mid‐March and the first generation in mid‐July. Females carrying egg sacs were also collected in both 2010 and 2016 to compare fecundity and egg diameter between the overwintering and first generations. Spiders were housed individually in opaque Plexiglas enclosures (5.0 × 5.0 × 7.5 cm, length × width × height) and were maintained in climatic chambers at 25 ± 0.5°C with 60 ± 10% RH and 14:10 hr light:dark cycle. To eliminate the possible changes of spider reflectance characteristics under the laboratory conditions, reflectance (%) of females and males was measured as soon as possible after the collections.

### 2.2 Measurement of spider reflectance

Reflectance was measured in adult female and male spiders of the overwintering (female: *N* = 36; male: *N* = 38) and the first (female: *N* = 34; male: *N* = 24) generations collected in 2010 using a method previously described by Li et al. (2008) with slight modifications. We used an Ocean Optic USB2000 spectrometer (Ocean Optics Inc.) and a DH2000 deuterium & tungsten halogen light source (Ocean Optics Inc.) to collect the spectral reflectance data. Approximately 5 min before obtaining measurements, an individual spider was anesthetized with CO_2_ and mounted on a fixed stage. Reflectance probes were held 2 mm above the body part to be measured, and at 90° to the surface. We measured reflectance in four body parts (dorsal carapace, lateral carapace, dorsal abdomen, and lateral abdomen) of each spider (Clark, Roberts, Rector, & Uetz, 2011). All reflectance measurements were performed in a dark room on a black matte surface. For each individual, five reflectance measurements were taken from each body part and these were averaged to obtain the mean reflectance of a body part. We also calculated the median for each 20 nm increment between 350 and 750 nm, creating a distribution of 21 values corresponding to the visual spectral range of wolf spiders (DeVoe, 1972).

### 2.3 Measurement of body size and fecundity

We also measured carapace width of the collected adult females and males to the nearest 0.025 mm in the overwintering (2010: female, *N* = 24; male, *N* = 20; 2016: female, *N* = 30; male, *N* = 20) and the first (2010: female, *N* = 20; male, *N* = 21; 2016: female, *N* = 20; male, *N* = 30) generations in both years using a binocular microscope. First, female spiders carrying egg sac were anesthetized using CO_2_ to remove the egg sac from the female. Then, the egg sacs of females from the overwintering (2010: *N* = 24; 2016: *N* = 30) and the first (2010: *N* = 20; 2016: *N* = 20) generations and 2 years were gently opened with a pin and the number of eggs in each egg sac was counted. Five eggs from each egg sac were then randomly selected to measure egg diameter using a binocular microscope.

### 2.4 Daily temperature data collection

The daily maximum and minimum temperatures during the study period (1 July 2009 to 31 July 2010, and 1 July 2015 to 31 July 2016) in Wuhan were provided by the Hubei Meteorological Bureau. In general, the temperature increases steadily from February to August and drops gradually from August to the following February. In both 2010 and 2016, the highest and lowest temperatures occurred in August and February, respectively (Figure S1).

### 2.5 Data analysis

Data were first checked for normality and were transformed whenever necessary to meet the assumption of normal distribution. Statistical significance of the differences between female and male reflectance (%) was analyzed by repeated‐measures two‐way ANOVA, with spider generations and body parts as fixed factors. The effects of spider generations on carapace width of females and males and egg diameter in both years were analyzed with two‐way ANOVA. Female fecundity was evaluated using ANCOVA with spider generations as fixed factor and female carapace width as a covariate as the latter strongly influences fecundity. Statistical analyses were performed with SPSS (version 13.0; SPSS Inc., Chicago, IL, USA).

## 3 RESULTS

### 3.1 Reflectance

**Figure 1:**
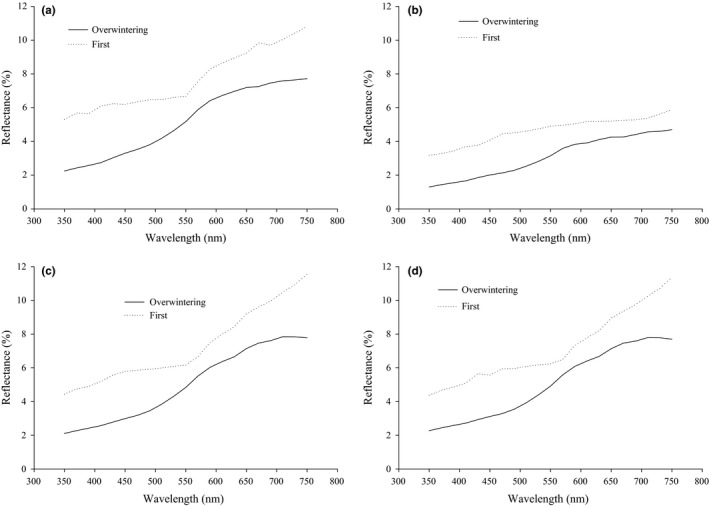
Reflectance of different body parts in the overwintering and the first generations of the female wolf spider, *Pardosa astrigera*, in 2010 [(a) dorsal carapace; (b) lateral carapace; (c) dorsal abdomen; (d) lateral abdomen]

In females, the carapace reflectance differed significantly between the two generations (*F*_1,66_ = 37.953, *p* < .001) and body parts (*F*_1,66_ = 59.85, *p* < .001), but was not significantly affected by the interaction between generations and body parts (*F*_1,66_ = 2.101, *p* = .152) (Figure 1). Reflectance was significantly lower in the overwintering generation than in the first generation (Figure 1a,b). Similarly, reflectance was higher in the dorsal carapace than the lateral carapace (Figure 1a,b). Moreover, reflectance in female abdomen was significantly affected by generations (*F*_1,66_ = 67.889, *p* < .001), but neither by body parts (*F*_1,66_ = 0.007, *p* = .936), nor by the generations/body parts interaction (*F*_1,66_ = 0.103, *p* = .749). Reflectance in female abdomen was significantly lower in spiders of the overwintering generation than the first generation (Figure 1c,d).

**Figure 2:**
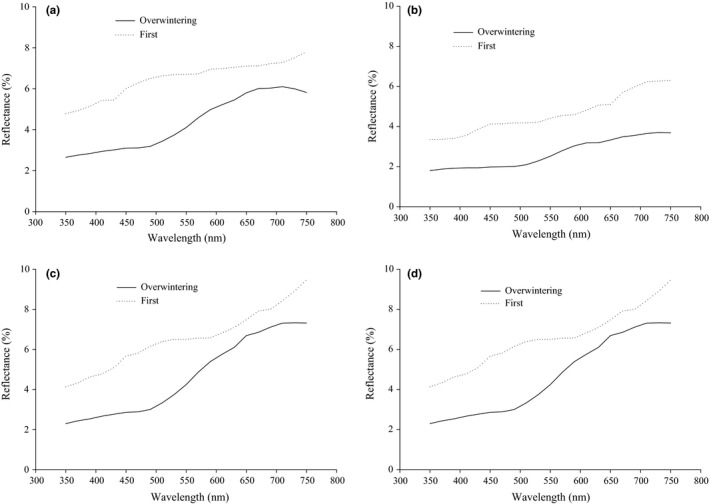
Reflectance of different body parts in the overwintering and the first generations of the male wolf spider, *Pardosa astrigera*, in 2010 [(a) dorsal carapace; (b) lateral carapace; (c) dorsal abdomen; (d) lateral abdomen]

In males, reflectance of the carapace was significantly affected by generations (*F*_1,58_ = 36.276, *p* < .001) and body parts (*F*_1,58_ = 33.451, *p* < .001), but not significantly affected by generations/body parts interaction (*F*_1,58_ = 0.112, *p* = .739) (Figure 2). Reflectance in male carapace was significantly lower in spiders of the overwintering generation than the first generation (Figure 2a,b). Similarly, reflectance was higher on the dorsal carapace than on the lateral carapace (Figure 2a,b). In the male abdomen, reflectance was significantly affected by generations (*F*_1,58_ = 44.325, *p* < .001), but not by body parts (*F*_1,58_ = 0.406, *p* = .526) or by the generations/body parts interaction (*F*_1,58_ = 0.001, *p* = .996). Moreover, reflectance in male abdomen was significantly lower in spiders of the overwintering generation than the first generation (Figure 2c,d).

### 3.2 Female and male carapace width

**Figure 3:**
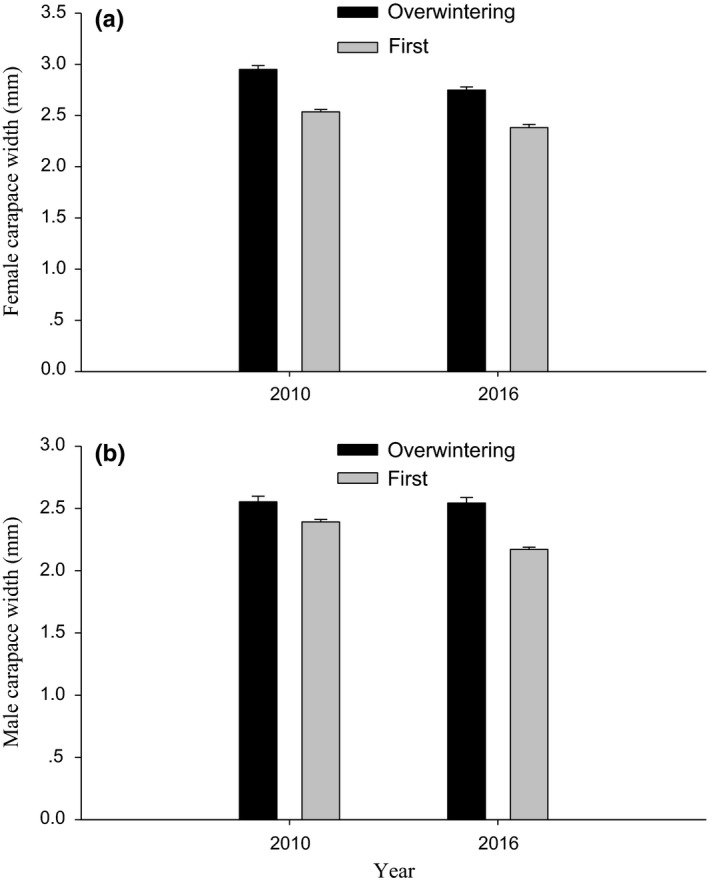
Differences in female (a) and male (b) carapace width (mean + *SE*) between the overwintering and the first generations of *Pardosa astrigera*

Width of the carapace in both females and males was significantly larger in the overwintering generation than the first generation in both years (Female: 2010, *F*_1,42_ = 79.929, *p* < .001; 2016, *F*_1,48_ = 66.541, *p* < .001; Figure 3a; Male: 2010, *F*_1,39_ = 11.078, *p* = .002; 2016, *F*_1,48_ = 78.590, *p* < .001; Figure 3b).

### 3.3 Fecundity

**Figure 4:**
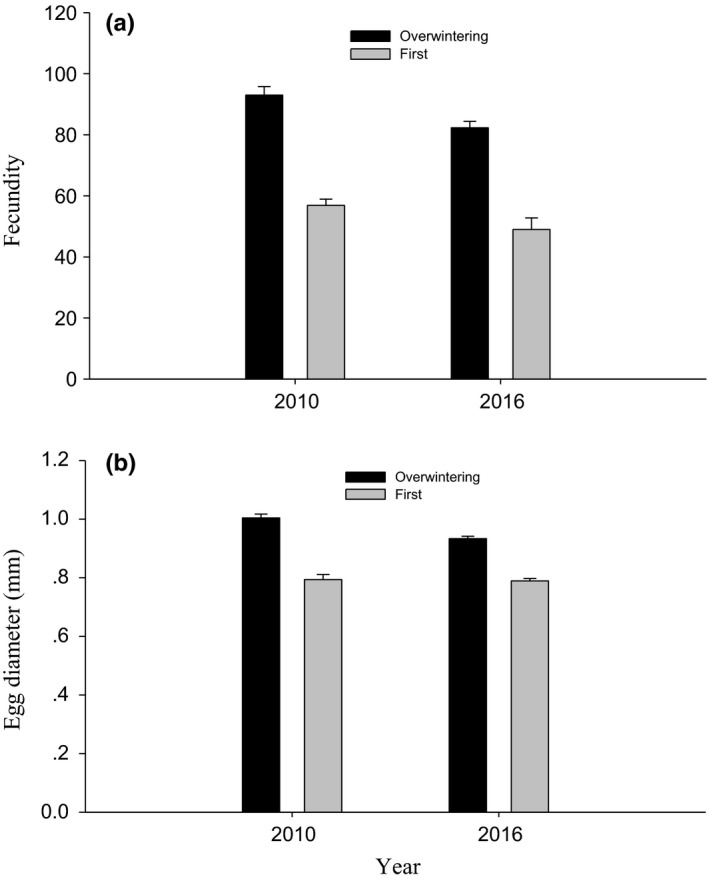
Differences in female fecundity (a) and egg size (b) (mean + *SE*) between the overwintering and the first generations of the wolf spider

Fecundity correlated positively with female carapace width (2010, *F*_1,41_ = 18.594, *p* < .001; 2016, *F*_1,47_ = 24.203, *p* < .001), and was markedly affected by generations (2010, *F*_1,41_ = 13.296, *p* = .001; 2016, *F*_1,47_ = 7.652, *p* = .008; Figure 4a). In both 2010 and 2016, fecundity was significantly higher in the overwintering generation than the first generation (Figure 4a).

### 3.4 Egg diameter

Egg diameter in the overwintering generation was also significantly larger than the first generation in both years (2010, *F*_1,42_ = 22.983, *p* < .001; 2016, *F*_1,48_ = 146.813, *p* < .001; Figure 4b).

## 4 DISCUSSION

In the present study, our results showed reflectance in both female and male spiders *P. astrigera* was significantly lower in the overwintering generation than in the first generation. Moreover, carapace width in both females and males was significantly larger in the overwintering generation than the first generation and fecundity was positively correlated with female carapace width. Lastly, fecundity was higher and egg diameter was larger in the overwintering generation than the first generation. These results show that *P. astrigera* exhibits substantial seasonal variations in adult body size, melanism, fecundity, and egg diameter.

In the present study, both female and male *P. astrigera* experiencing the cold season (overwintering generation) were darker than those in the warm season (first generation). These results are in agreement with the thermal melanism hypothesis, which indicates that darker individuals have an advantage in cold environments because of their ability to absorb heat from solar radiation and become active. Conversely, in the first generation, individuals with lighter body color could avoid the risk of overheating, which is an advantage in warmer conditions. Similar results have been widely reported across taxa (Clusella‐Trullas et al., 2007). Considering that Wuhan winters are not only cold but also dry, it is likely that body melanism also plays a role in desiccation resistance in the wolf spider. Several studies in insects have shown that body melanisation is positively linked with desiccation resistance (Daniels et al., 2012; De Souza et al., 2017; Gibbs et al., 2003; Parkash, Rajpurohit, et al., 2008; Parkash, Ramniwas, et al., 2008; Parkash et al., 2010; Rajpurohit et al., 2008; Ramniwas et al., 2013). However, it should be noted that thermal melanism and melanism desiccation are not mutually exclusive. Considering the darker body of the overwintering spider and the light background, we could rule out the hypothesis of cryptic coloration (Clark et al., 2011). Due to low risk of ultraviolet radiation and disease spread in winter, the possibilities that the melanism of the overwintering spider to ultraviolet protection and disease resistance could be ruled out (Majerus, 1998; Reguera et al., 2014).

In addition, our results indicate that the reflectance (%) of dorsal carapace is significantly higher than the lateral carapace in both female and male *P. astrigera*. These results are consistent with our visual observation that the dorsal carapace was lighter than the lateral carapace. Similar results were reported in the wolf spider, *Schizocosa ocreata* (Clark et al., 2011).

In addition to the seasonal variations in body melanism in the wolf spider *P. astrigera*, body sizes of both females and males of the overwintering generation were markedly larger than the first generation. These results are consistent with those reported by Iida et al. (2016). It is widely reported that adult body size can be substantially affected by temperature during the early stage of development (Atkinson, 1994; Noriyuki, Kishi, & Nishida, 2010; Ray, 1960). In general, offspring that develop at lower temperatures develop into larger adults and vice versa (Atkinson, 1994; Ray, 1960), likely because larger body with smaller specific surface area favors heat loss avoidance. It seems that the larger body sizes of overwintering *P. astrigera* are adaptations to the low temperature. Such effects of differential temperatures on adult body size may explain the different patterns of body size in *P*. *astrigera* during different seasons. Similar results have been widely reported in arthropods (Atkinson, 1994; Ray, 1960). For example, in the butterfly, *Ypthima multistriata*, overwintered larvae grew into large first‐generation adults in the spring when the temperature is relatively low, whereas second‐generation larvae that developed during the hot summer grew into smaller adults (Noriyuki et al., 2010). Besides temperature, insect body size is also markedly affected by photoperiod, that is, adults grow larger under long photophase relative to those under short photophase. For example, the head and pronotum widths of *Halyomorpha halys* (Stål) were significantly reduced under a short photophase (Niva & Takeda, 2003). In addition, *Dolycoris baccarum* (L.) and *Piezodorus guildinii* adults reared at 25°C under long photophase conditions were larger than those under short photophase (Nakamura, 2002; Zerbino et al., 2014). Considering that overwintering *P. astrigera* with large body size experience gradually decreased photoperiod and temperature, it appears that the body size of *P. astrigera* is not controlled by photoperiod. These results are consistent with the results from *Nezara viridula*, which had larger adults in naturally decreasing photoperiod and temperature when they had a longer development time (Musolin, Tougou, & Fujisaki, 2010). Given a positive relationship between photoperiod and temperature in nature, it seems likely that body size in arthropods is mediated by these two factors. In some species, the effects of temperature override photoperiod (Iida et al., 2016; Musolin et al., 2010). However, in other species, body size is significantly affected by photoperiod than by temperature (Nakamura, 2002; Niva & Takeda, 2003; Zerbino et al., 2014, 2015). The contrasting mechanisms regulating the interaction between temperature, photoperiod, and body size needs further investigation.

It is well known that female body size positively correlates with fecundity in insects (Honěk, 1993). Our current results along with others (Iida et al., 2016) are consistent with this paradigm. However, our results indicate that fecundity is not only affected by female body size but also by generations. Given the positive relationship between fecundity and female body size of the wolf spider *P. astrigera*, it seems likely that higher fecundity may be the by‐product of the larger female body size. Besides being positively impacted by female body size, fecundity is also affected by generations, that is, the fecundity of overwintering females is significantly higher than the first‐generation females. Because the resources and/or energy of the overwintering females accumulate during their offspring stage, it is predicted that there is a tradeoff between female reproduction and survival, and between their current reproduction and future reproduction. Moreover, as the females lack prey and shelter, as well as incur higher predatory risk in early spring, it seems likely that overwintering females may allocate most resources to current reproductive output rather than future reproduction and/or survival.

In the present study, *P. astrigera* females of the overwintering generation produced significantly larger eggs than the first generation. It is presumed that egg size is subject to selection, because it has substantial fitness effects on progeny (Pöykkö & Mänttäri, 2012). Because larger eggs yield larger offspring, mothers often lay larger eggs under stress conditions (Fischer, Bot, et al., 2003; Fischer, Brakefield, et al., 2003; Fischer et al., 2004, 2006; Hassall, Walters, Telfer, & Hassall, 2006). Iida et al. (2016) found that seasonal variations in the body sizes of *P. astrigera* spiderlings occur, that is, significant negative correlations were found between temperature and cephalothorax and abdomen widths of spiderlings. Although they did not directly measure the egg size across seasons, it seems likely that the larger spiderlings result from larger eggs. Compared with the spiderlings hatched from smaller eggs, it is generally accepted that the spiderlings hatched from larger eggs have higher resistance to environmental stresses, such as intraspecific competition, starvation, desiccation, and low temperature (Fox & Czesak, 2000). In East Asia, the month of March is cold and dry with shortage of prey. In these conditions, the larger spiderlings produced by the overwintering females may be adaptive to the stress environment (Iida et al., 2016). Similar results have been reported across arthropoda. For example, larger first‐instar larvae of the coleopteran parasitoid, *Aleochara bilineata*, are more active, survive longer, and parasitize their host more rapidly (Boivin & Gauvin, 2009). Offspring from larger eggs also develop faster and grow into larger adults in the seed beetle, *Callosobruchus maculatus* (Fox, 1994). In the wolf spider, *Hogna helluo*, starvation tolerance and feeding performance of offspring correlate positively to offspring size (Walker, Rypstra, & Marshall, 2003). In the wolf spider, *P. pseudoannulata*, spiderling cephalothorax width strongly affects hunting ability and abdomen width greatly affects starvation tolerance (Iida, 2005). Several studies have suggested that increased egg size may be adaptive at low temperatures, with larger eggs having higher hatch rates, higher survival to adulthood, and shorter larval development times (Fischer, Bot, et al., 2003; Fischer, Brakefield, et al., 2003; Fischer et al., 2004, 2006; Hassall et al., 2006).

## CONFLICT OF INTEREST

The authors declare no conflict of interest.

## AUTHOR CONTRIBUTIONS

XGJ and JC designed the experiment. JJY, QJW, and RX performed the experiment. XGJ and JPZ analyzed the data. XGJ wrote the paper. All authors read and approved the manuscript.
