# Supplementary material for: Seasonal variations in body melanism and size of the wolf spider Pardosa astrigera (Araneae: Lycosidae)
Source: Ecol Evol. 2018 Apr 2;8(8):4352–9. doi: 10.1002/ece3.3988 (PMC5916282; doi:10.1002/ece3.3988)
Supplement: Supplementary file 1 [file ECE3-8-4352-s001.doc]

**Figure S1. Yang et al.**

Figure S1．Daily air temperature dynamic in Wuhan City, Hubei Province, China (A. July 1, 2009 to July 31, 2010; B. July 1, 2015 to July 31, 2016)
